# Wisepatern mastopexy for natural breast ptosis symmetrization after giant lipoma excision: Case report

**DOI:** 10.1016/j.ijscr.2023.108532

**Published:** 2023-07-21

**Authors:** Kristanto Yuli Yarso, Monica Bellynda, Erwin Aritama Ismail, Muhammad Rizki Kamil

**Affiliations:** a Division of Oncology Surgery, Department of Surgery, Universitas Sebelas Maret - Dr. Moewardi General Hospital Surakarta, Indonesia; b Department of Surgery, Universitas Sebelas Maret - Dr. Moewardi General Hospital Surakarta, Indonesia

**Keywords:** Wisepatern, Mastopexy, Giant breast lipoma, Case report

## Abstract

**Introduction and importance:**

Giant breast lipoma is an uncommon benign tumor that develops in the breast parenchyma. Wisepatern technique involves lifting the skin in both vertical and horizontal directions to raise and reshape the breasts into a less ptotic shape.

**Case presentation:**

A 40-year-old woman came to the Surgical Oncology Polyclinic with the breast size is asymmetric, the right is 4× larger than the left. The patient's request for a tumor removal procedure with a symmetrical approach on the right breast alone, we opted for a Wisepatern surgical technique. Residual skin tissue was discovered and de-epithelialization was performed to remove it, but it was not discarded and instead inserted into the breast cavity to create the effect of a mass filling empty areas left after tumor removal.

**Clinical discussion:**

The Wisepatern technique is highly preferred due to its versatility, ease of execution, and ability to achieve consistent outcomes in mastopexy and breast reductions. It effectively addresses excess skin, avoids large dog-ears and longer scars, and allows for a more natural-looking appearance by utilizing a shortened vertical scar and partial subpectoral pocket for implant positioning.

**Conclusion:**

Mastopexy is a surgical procedure that can effectively treat giant breast lipoma by removing the lipoma and reshaping the breast tissue. However, it is important for patients to be fully informed about the risks and benefits of the procedure and to undergo appropriate follow-up to ensure a successful outcome.

## Highlights

• Giant breast lipoma was successfully treated using a Wise pattern surgical technique for removal and breast reshaping.
• The patient's request for a tumor removal procedure with a symmetrical approach on the right breast alone poses a considerable challenge, which is frequently encountered among female patients in Indonesia.
• The Wise pattern technique, involving lifting the skin in both vertical and horizontal directions, was employed to raise and reshape the affected breast, resulting in a less ptotic shape.
• Following complete removal of the tumor, the patient's seated position was adjusted to match the height of the nipple with the contralateral healthy breast, and residual skin tissue was repurposed to fill empty areas left after tumor removal.
• The importance of patient education, informed consent, and appropriate post-operative follow-up for successful outcomes of Mastopexy procedure

## 1 Introduction

Giant breast lipoma is an uncommon benign tumor that develops in the breast parenchyma, characterized by a slow-growing mass that can cause breast enlargement and distortion, and in some cases, discomfort or pain [1]. The etiology of this condition is not well understood, but it is believed to be related to the proliferation of mature adipocytes [2]. Although there are only a few reported cases of giant breast lipoma in the medical literature, it can have significant cosmetic and psychological effects on affected individuals, and surgical excision is often necessary for definitive management [3]. Diagnosis of giant breast lipoma usually involves clinical examination, imaging studies, and biopsy.

Wisepatern is the first mastopexy technique presented by Wise in 1956. This technique involves lifting the skin in both vertical and horizontal directions to raise and reshape the breasts into a less ptotic shape. The Wisepatern has previously been applied to minimize skin during skin-sparing mastectomies, with the goal of eliminating any excess skin that could lead to unwanted side effects such as skin wrinkling, poor breast shape, implant issues, and an increased likelihood of fluid accumulation [4]. The advantage of this technique is using the remaining skin tissue that we will throw away into tissue that will fill the void after the removal of the tumor. Of course, it is very far compared to implants that fill up to 250-300 cc. We use this technique to carry out the symmetry of patients in Indonesia. Here we report a case of Wisepatern mastopexy for natural breast ptosis symmetrization in our Hospital. The aim of this production is to highlights Wisepatern mastopexy. This serial cases have been reported in line with the SCARE criteria [5].

## 2 Case report

**Fig. 1:**
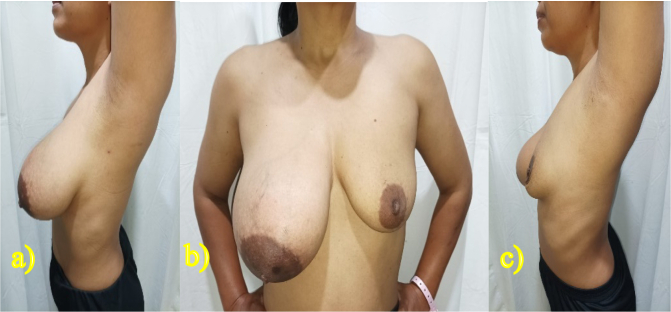
Physical examination showed asymmetrical breast, the right breast four time bigger larger than the left. a) right-sided view, b) front view, and c) left-sided view.

**Fig. 2:**
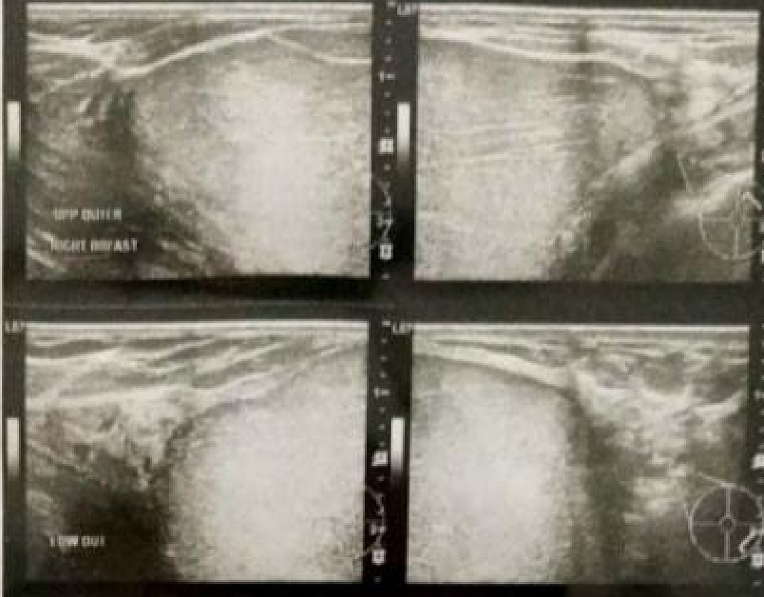
Ultrasound examination showed homogeneous solid lesion, oval in shape with firm boundaries, encapsulated in the right breast quadrant.

A 40-year-old woman came to the Surgical Oncology Polyclinic with complaints of a lump in her right breast, which had enlarged slowly within 5 years since breastfeeding her second child. The lump does not feel hard, is painful, feels warm, and there is no change in the menstrual cycle. The patient had no history of malignancy and no family history of breast cancer. The patient had no surgical history, no drug history, and no any medical history relevant to breast disease. The patient is Javanese housewife, non-smoker, with BMI about 23.5 kg/m^2^. On physical examination the breast size is asymmetric, the right is 4× larger than the left with size 10 × 10 × 7 cm (Fig. 1), feels soft springy with the consistency of fat, firm boundaries, no tenderness, and skin wounds. The diameter of the right areola is enlarged to 9 cm, there is no nipple discharge. No lumps were found in the armpits. Breast ultrasound examination showed homogeneous solid lesion, oval in shape with firm boundaries, encapsulated in the right breast quadrant (Fig. 2). Biopsy examination showed lobulated adipose tissue with minimal connective tissue stroma, the impression of lipoma. The patient declined to undergo a symmetrical mastopexy procedure accompanied by bilateral implants due to financial constraints and an aversion to unnecessary surgery on the healthy breast, which is frequently encountered among female patients in Indonesia. As a solution, we opted for a Wisepatern surgical technique, whereby the remaining tissue is not excised but instead de-epithelialized and inserted into the breast to fill the void left by the tumor's removal. The surgery was performed by an experienced oncology surgeon with over 5 years of experience in oncoplastic breast reconstruction.

**Fig. 3:**
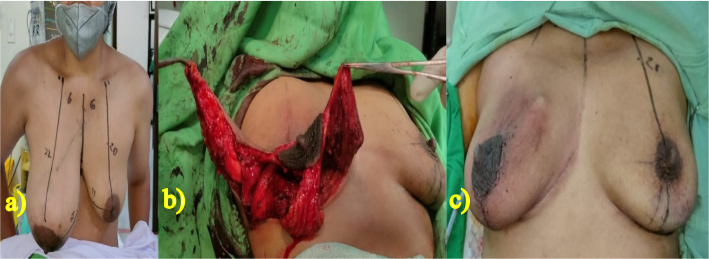
Durante operation. a) The marked with traditional Wisepatern, b) incision following the pattern, and c) after two layers of suturing.

**Fig. 4:**
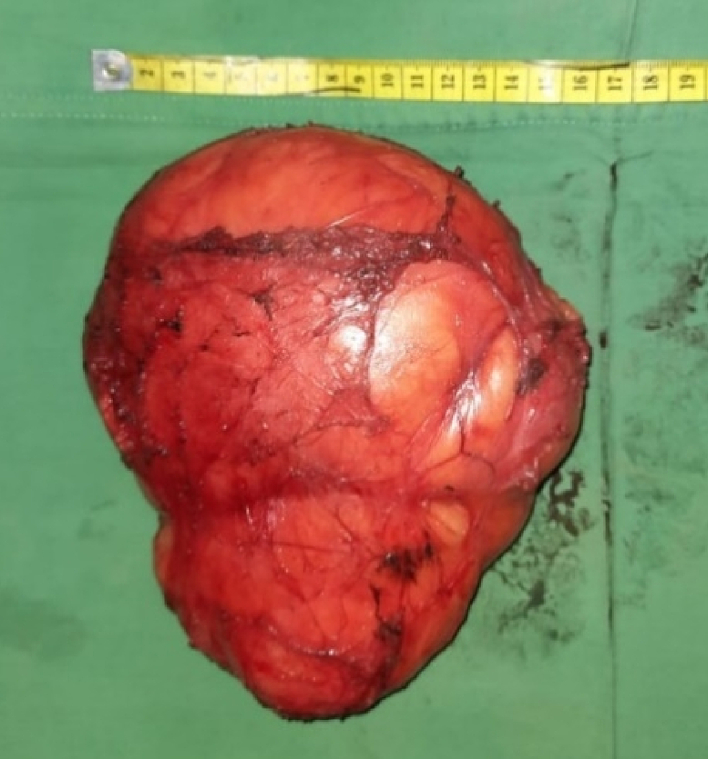
The tumor mass was successfully removed sized 16,5 × 15,5 × 15 cm.

The patient's symmetry and measurements were evaluated relative to the healthy side of the contralateral breast, while the patient was seated with arms at their sides. The patient was marked for a traditional Wisepatern skin reduction while standing, as if undergoing a standard breast reduction procedure. Informed consent to the patients, site-marking, and prophylaxis antibiotics were done before the procedure under the anesthesia. Following general anesthesia, an incision was made and the tumor was removed along the previously marked design line with 30 Hz electrosurgical frequency. Once the tumor was completely removed without pedicle reduction, then the patient was seated at a 45-degree angle to match the height of the nipple with the contralateral healthy breast. A minor revision of the initial design was made, and an incision was created accordingly. Residual skin tissue was discovered and de-epithelialization was performed to remove it, but it was not discarded and instead inserted into the breast cavity to create the effect of a mass filling empty areas left after tumor removal (Fig. 3). An 18G drain was inserted, and two layers of suturing for subcutaneous and intradermal tissue were performed in stages using 5.0 absorbable thread. The tumor was completely removed measured to be 16,5 × 15,5 × 15 cm, encapsulated with intact yellow-brownish capsule and 1.400 g of mass (Fig. 4). The surgery lasted for 3 h, and after the operation, the patient did not require a blood transfusion. The patient received mefenamic acid (500 mg orally three times a day) for postoperative pain relief.

**Fig. 5:**
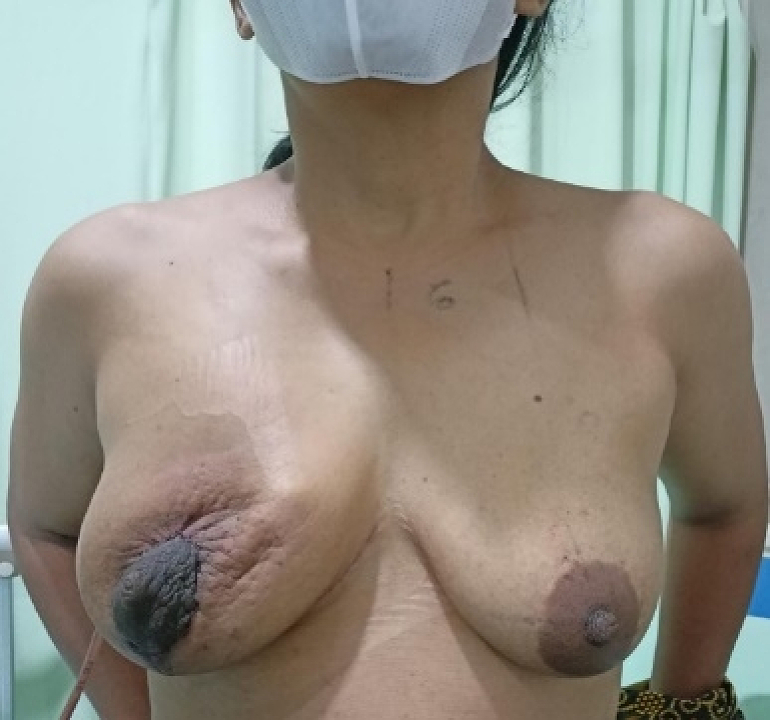
2 days after the procedure, drain will be removed and patient will be discharged.

**Fig. 6:**
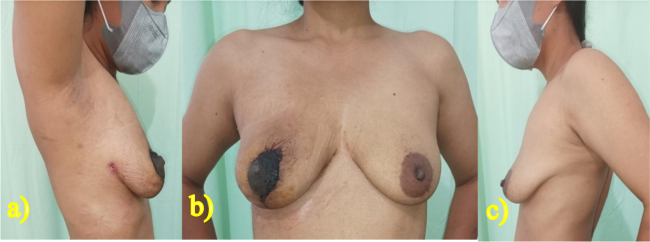
1 month follow up. a) Right-sided view, b) front view, and c) left-sided view.

**Fig. 7:**
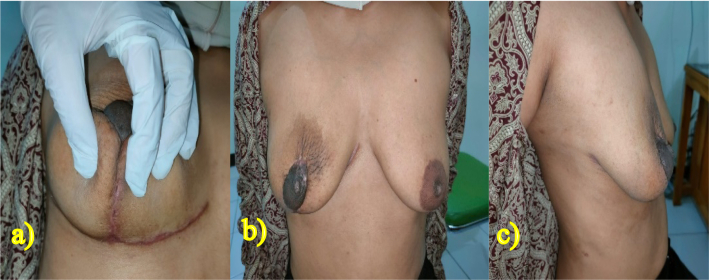
3 months follow up. a) Bottom view, b) front view, and c) left-sided view.

**Fig. 8:**
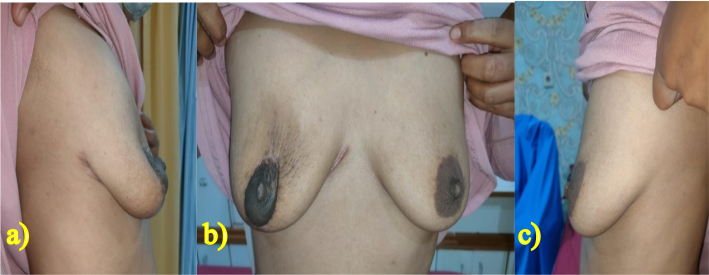
6 months follow up. a) Right-sided view, b) front view, and c) left-sided view.

For evaluating the fluid production after 24 h of surgery, the drain was removed, and the fluid found to be 100 ml. On the second day of treatment, the patient was discharged from the hospital (Fig. 5). The postoperative period was uneventful, and no complications such as hematoma, incision complications, infections, or neurological issues were observed. There is no any complications relating to surgery and anesthesia. However, marginal epidermal necrosis occurred within the first week, which subsequently improved after the initiation of epidermal growth in the first month. The patient was monitored at the hospital in the 1st, 3rd, and 6th months, and there was no indication of infection at the surgical site (Fig. 6, Fig. 7, Fig. 8). The patient reported being satisfied with the operation's outcome.

## 3 Discussion

Lipoma is the most common type of soft tissue neoplasm arising from mesenchymal tissue, with a prevalence rate of 2.3 cases per 1000 individuals. It typically develops during the fifth and sixth decades of life. It can occur in any parts of body, most commonly found in the arm, shoulder, back, legs, and face and estimated about 20 % in the chest wall [6,7]. The literature does not provide a precise estimation of the occurrence rate of breast lipoma. It is classified as a giant lipoma if its size exceeds 10 cm in any one dimension or weighs at least 1000 g [2].

Giant breast lipomas can be difficult to diagnose, as they may be mistaken for other breast masses, such as fibroadenomas or cysts. Diagnosis of giant breast lipoma usually involves clinical examination, imaging studies, and biopsy. Clinical criteria which involved identifying a tumor that was well-defined, smooth, and possibly slightly lobulated. The tumor was typically soft or semi-firm to the touch and was mobile when palpated. In cases of large or deep tumors, physical examination may not provide much diagnostic value [8,9]. On mammography, a radiolucent nodule surrounded by a thin radiopaque capsule that may include ring-like calcifications owing to fat necrosis. Biopsy finding shows huge soft consistency, irregular surface, and yellow-colored tissue and Cut sections showed adipose tissue without evidence of atypia or malignant change [10].

Following the removal of the tumor, our objective is to achieve a symmetric and proportional outcome without using silicone implants. There are more than one hundred techniques for mastopexy with or without the use of implants, which have been documented in the literature [11]. We prefer the Wisepatern technique because its versatility, ease of execution, and can achieve consistent outcomes. It is the most widely used technique for both mastopexy and breast reductions. The procedure also addresses excessive unwanted skin often plaguing a ptotic breast and excises it as part of the procedure [12,13]. This procedure allows surgeons to avoid making a horizontal incision across the middle of the breast, which would result in large dog-ears that would need to be removed, leaving a longer scar. The shortened vertical scar of the inverted T pattern can be partly concealed by a reconstruction of the nipple and areola [14]. Furthermore, the Wisepatern technique permits the implant to be positioned in a partial subpectoral pocket, which can offer extra support and a more natural-looking appearance to the breast [15].

One of the primary concerns is the risk of flap necrosis, particularly at the junction of the horizontal and vertical limbs. Some studies have reported other complications such as delayed wound healing, infection, seroma, and hematoma [16]. Smoking and BMI more than 35 also been associated with an increased risk of skin loss and implant failure [17]. The disadvantages of this procedure are long scar in the inframammary fold (IMF), wound dehiscence, healing problems in the T-corner, and elongation of the nipple IMF distance over time. Minor and major complication rates have been reported, including venous insufficiency, partial areola necrosis, wound-opening problems, and dog-ear deformities [18]. Complication in this patient were treated conservatively and resolved successfully. Patients who have a tendency towards keloid formation or hypertrophic scarring should be taken into consideration prior to undergoing this procedure [19].

Other modalities are known to be considered for the treatment of giant breast lipoma. Liposuction as an optional management could has better cosmetic result because it allows the incision to be placed in less visible area. However, this related to high risk of recurrence, hematoma formation, dimpling, sensory and pigmentation disturbances. Therefore, liposuction is unable to address the significant empty area that occurs after the removal of a breast tumor, as well as the surplus of skin that has been stretched by the tumor [10,19]. To achieve symmetrical breasts, the option of Contralateral Reduction Mastopexy can be performed gradually during lipoma removal surgery. The use of autologous tissue in breast reconstruction can minimize healing time and hospital stay, thereby improving the quality of life [[20], [21]]. However, we take into consideration the patient's preferences. The patient declined the procedure as she refused to have surgery on her healthy breast. The use of breast implants can be considered as an alternative option since it eliminates the need for surgery on the healthy breast. However, the patient declined due to financial concerns.

## 4 Conclusion

Mastopexy is a surgical procedure that can effectively treat giant breast lipoma by removing the lipoma and reshaping the breast tissue. However, it is important for patients to be fully informed about the risks and benefits of the procedure and to undergo appropriate follow-up to ensure a successful outcome. Further research is needed to determine the long-term outcomes of mastopexy for giant breast lipoma and evaluating the possibility of lipoma relapse.

## Ethical approval

Ethical approval is exempt/waived at our institution. The study protocol was approved by Research Ethics Committee Sebelas Maret University on May, 26th 2023 with registration number: 115/UN27.06.11/KEP/ECC/2023. Titled: “Wisepatern Mastopexy for Natural Breast Ptosis Symmetrization after Giant Lipoma Excision: Case Report (Wisepatern Mastopeksi untuk Simetrisasi Ptosis Payudara Alami setelah Eksisi Giant Lipoma: Laporan Kasus)”.

## Financial disclosure

The authors declared that this study has received no financial support.

## Source of support

This article is supported by Departments of Oncology Surgery, Medical Faculty, Sebelas Maret University, Indonesia.

## Patient consent for minors

Written informed consent was obtained from the patients for publication of this case report and accompanying images. A copy of the written consent is available for review by the Editor-in-Chief of this journal on request.

## Credit authorship contribution statement

Kristanto Yuli Yarso: study conception and design, analysis and interpretation of results, reviewed the results and approved the final version of the manuscript; Monica Bellynda: study conception and design, data collection, analysis and interpretation of results, draft manuscript preparation, reviewed the results and approved the final version of the manuscript; Erwin Aritama Ismail: study conception and design, data collection, analysis and interpretation of results, draft manuscript preparation, reviewed the results and approved the final version of the manuscript Muhammad Rizki Kamil: analysis and interpretation of results, draft manuscript preparation, reviewed the results and approved the final version of the manuscript.

## Research registration

N/A

## Declaration of competing interest

The authors declare no conflict of interest.
